# A comparison between the measurement of quantum spatial correlations using qCMOS photon-number resolving and electron multiplying CCD camera technologies

**DOI:** 10.1038/s41598-024-64674-5

**Published:** 2024-06-26

**Authors:** K. Roberts, O. Wolley, T. Gregory, M. J. Padgett

**Affiliations:** School of Physics and Astronomy, University of Glasgow, Glasgow, UK

**Keywords:** Optical physics, Quantum optics

## Abstract

Cameras with single-photon sensitivities can be used to measure the spatial correlations between the photon-pairs that are produced by parametric down-conversion. Even when pumped by a single-mode laser, the signal and idler photons are typically distributed over several thousand spatial modes yet strongly correlated with each other in their position and anti-correlated in their transverse momentum. These spatial correlations enable applications in imaging, sensing, communication, and optical processing. Here we show that, using a photon-number resolving camera, spatial correlations can be observed after only a few 10s of seconds of measurement time, thereby demonstrating comparable performance with previous single photon sensitive camera technologies but with the additional capability to resolve photon-number. Consequently, these photon-number resolving technologies are likely to find wide use in quantum, low-light, imaging systems.

## Introduction

Quantum enhanced imaging can surpass classical limits by exploiting the spatial correlations between photon-pairs generated by spontaneous parametric down-conversion^1–5^. To observe these spatial correlations requires a detector technology that is both sensitive to single photons and has a low enough noise floor to avoid too many false positives. Single-photon sensitive, single-pixel (single-element) detectors are common place. However, using only a single-pixel detector in imaging schemes leads to slow acquisition rates since the pixel needs to be spatially scanned over the field of view, introducing sampling losses, meaning that many photons go undetected. Single-photon sensitive detector arrays remove this sampling loss and hence reduce acquisition times through parallelised detection of the entangled photon-pairs. Such detector array technology in the form of electron multiplying CCDs (EMCCD), intensified CCDs (ICCD), single-photon avalanche detectors (SPAD) cameras have been, and continue to be, used for quantum imaging schemes but are limited in their ability to accurately count the number of photons detected in each pixel beyond either a 0 or $\ge 1$ photons^6,7^. This binary output restricts the data throughput of the camera system and therefore limits the overall speed of quantum imaging schemes. The recent development of low-noise detector technologies capable of photon-counting will allow for greater overall efficiency in performing quantum imaging experiments. The performance of these detector arrays can be characterised by the time required to observe the spatial correlation between the down-converted signal and idler photons. EMCCD camera detectors have previously been used to characterise the correlations between entangled photon-pairs^8,9^ and even used to demonstrate the EPR paradox in a single pair of frames from two separated cameras^10^. Yet, while it is possible to observe these spatial correlations in a single pair of frames, to characterise the strength of the correlations, such as when trying to position the array detector in the optimum plane, a greater number of frames are required. This can lead to an increased acquisition time, especially when using a camera sensor maintained at $-90^{\circ }C$ to minimise detector noise which requires temperature stabilisation.

In Spontaneous Parametric Down-Conversion (SPDC) the non-linear process results in a pump photon being absorbed, creating a pair of signal and idler photons. This process is constrained by conservation laws of energy and momentum and as such the pair of generated photons are correlated in their position and anti-correlated in their transverse momentum^11^, as observed in the image plane and far-field planes of the down-conversion crystal respectively. The photon-pairs generated in the low-gain regime (SPDC) are independent of one another and are also an example of the position-momentum entanglement presented in the original EPR paradox^8–10,12,13^. As such, the development of detectors capable of detecting the single photons that form these entangled photon pairs has led to the application of a number of detector types for use in quantum imaging experiments^5^.

As mentioned above, initial experiments to detect SPDC and characterise the spatial correlations were implemented using pinhole scanning techniques and single-pixel detectors^14–16^. The need for a spatially selective scanning process introduces sampling loss and leads to long acquisition times. By contrast, array detectors are capable of simultaneously capturing over the full spatial extent of the photon distribution giving a speed up in proportion to the number of possible spatial positions. An early example of using a detector array to measure spatial correlations between photon-pairs in down-converted light utilised an ICCD camera^17^. ICCD cameras employ an image intensifier comprising a photocathode to convert single photons into single electrons, a microchannel plate to multiply the electrons, and a phosphor screen to convert these electrons into many photons in order to amplify the optical signal incident upon the camera, enabling a single-photon to be observed above the noise of the detector. Furthermore, the intensifier can be turned on only for a gate-time much shorter than the CCD exposure to prevent amplification of background noise^18^. Subsequent experiments operated in the high-gain regime, parametric down-conversion, (PDC) where the higher intensity enabled standard CCD detectors to be utilised to observe spatial correlations in the far-field intensity pattern of PDC beams^19^. The sub-shot-noise nature of these intensity correlations in PDC beams was then subsequently demonstrated also using a CCD camera^20,21^. In the low-gain regime, CCD cameras have also been used for the sub-shot-noise imaging of a weakly absorbing object^22,23^. However, this low-gain regime is typically the regime in which EMCCD cameras are utilised in quantum optics experiments due to their high EM-gain that amplifies low signals thereby giving single-photon sensitivity. In EMCCD cameras the gain is on-chip and as such any background and detector noise is amplified alongside the signal by the high EM-gain readout^24,25^. EMCCD detectors have a greater efficiency ($\sim 60 \%$ after thresholding) when compared to ICCDs ($\sim 20 \%$ after thresholding) but lack the capacity to time-gate the detection below the CCD exposure time^5^. Nevertheless, EMCCD cameras have been used in a large number of quantum optics experiments ranging measuring the sub-shot-noise nature of SPDC light^25–30^ to using these correlations to demonstrate the EPR paradox^8–10,31,32^, and imaging Hong-Ou-Mandel interference^33^.

Beyond demonstrations of fundamental quantum mechanics, EMCCD, ICCD, and scientific CCD camera technology have been used extensively to perform quantum imaging using correlated photons, demonstrating a range of improvements over classical techniques including those to overcome background noise^34–40^, improve image resolution^41,42^, ghost imaging^43–45^, sub shot-noise imaging^23,26,28^, imaging through scattering media^46,47^, and phase imaging^48^. There are also new array detector technologies being developed which have new capabilities regarding their time resolution, detector noise, and photon-number counting. The time resolution for the CCD detectors is limited by the exposure and readout time per frame which is of the order $\sim 10 \text { ms}$. This gives no ability to preferentially select photon-pairs and reject noise by arrival time. In the case of an ICCD camera the gate-time of the intensifier (and therefore the minimum time resolution) is of order $\sim 10 \text { ns}$. However, the overall throughput is still limited due to the CCD readout time. SPAD array cameras with time tagging capabilities have time resolution of the order $\sim 100 \text { ps}$ and can operate at very high readout rates^49–54^. Despite their excellent temporal resolution, SPAD based image sensors typically have a low fill-factor and therefore a low overall quantum efficiency and often only a small number of pixels^55^. These limitations notwithstanding, SPAD array detectors have been used to observe correlations of sufficient strength to demonstrate the EPR paradox^56–58^, perform quantum imaging in a number of different experimental configurations^39,59–61^, and implement quantum algorithms using metasurfaces^62^. Superconducting nanowire single-photon array detectors (SNSPD) have also been developed with high quantum efficiency, timing resolution, a broader spectral bandwidth than silicon based detectors, and low-noise. SNSPDs are limited by a low fill-factor of a few % (as with SPADs arrays) and also require cryogenic cooling, thereby potentially limiting deployment beyond lab applications^63^. SNSPDs detectors are increasing in their available pixel resolution^64^ as multiplexed readout methods are developed^65^. This will serve to improve the suitability of SNSPDs for applications in quantum imaging thereby extending their use beyond single pixel detector^66,67^ and single pixel imaging applications^68^. An event driven, single-photon sensitive, time-tagging camera based on CMOS technology incorporating an intensifier has also been developed as an alternative to ICCD and SPAD array detectors^69,70^. This form of detector has also been used to characterise spatial correlations and entanglement^71–78^ and perform quantum imaging^79,80^.

The camera technologies described above enabled detection of either many-photon intensity correlations or photon pair correlations to study quantum phenomena and deliver imaging enhancements. There is, however, a gap in the technology, which combines a high fill-factor array with a high quantum efficiency, with the ability to resolve the number of photons in each detector pixel. Addressing this gap, the latest qCMOS imaging sensors have single-photon-sensitivity with a sub-photon readout-noise and hence and photon-counting capabilities^81–83^. We have previously demonstrated the use of one such photon number resolving array detector to perform a quantum imaging experiment to improve image contrast^84^. In that experiment the detector was located in the image plane of the down-conversion crystal and the two-photon events were preferentially selected to construct an image. Using this camera, we were able to demonstrate an improved image contrast when illuminating the object using a spatially correlated light source comprising of SPDC photon-pairs compared to illuminating using a non photon-pair source. This improvement in image contrast was achieved by selecting the two-photon events while rejecting one-photon events. This selection criteria serves to preferentially select the photon-pairs, which are correlated in their generation area, as two-photon events that are detected in the same pixel of the camera positioned in the image plane of the downconversion crystal. The one-photon events correspond to single-photons, background light, and sensor noise and so are rejected thereby improving the image contrast. The detector used is capable of photon-number-resolving capability beyond 0, 1, or 2 photon events and is capable of detecting up to 200 photons in the low-noise high gain photon number resolving readout mode^85^. As such, characterising higher order SPDC generation which entails the detection of multiple photon-pairs in a single pixel is a potential avenue of research that could be conducted with such array detectors.

In this present work we characterise the performance of one of these photon number resolving detector arrays, a Hamamatsu ORCA-Quest qCMOS detector, to detect the spatially correlated photon-pairs generated by SPDC. The number of frames required to identify the correlation peak of a given signal to noise ratio is assessed for a range of levels of pixel occupancy equivalent to an average between 0 and $\sim 16$ photon events per pixel per frame. The correlation peak is assessed based on the correlations in the photon number counts between anti-correlated pixel positions in the two spatially separated downconversion beams present in the far-field of our source cut for type-II downconversion. This performance is then compared to that of an Andor Ultra 897 EMCCD detector, for which we assess the correlation peak based both on binarised frames corresponding to 0 or $>1$ photon events and non-binarised analogue output. Characterising this new photon-counting detector type and comparing to already existing technologies is a useful step in assessing its use within quantum enhanced imaging and other multi-pixel quantum applications.

## Experimental setup

**Figure 1:**
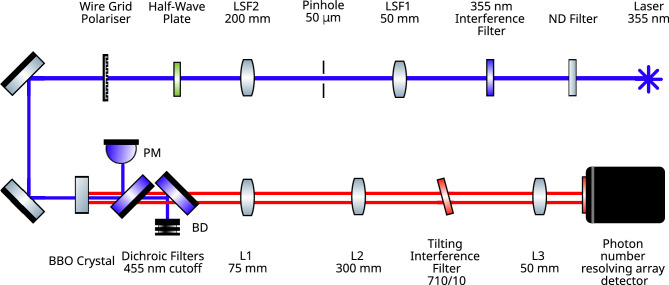
Schematic of the experimental setup. A $355 \text { nm}$ laser pumps a BBO crystal cut for type-II degenerate down-conversion to generate down-converted photon-pairs at $710 \text { nm}$. Lenses $L_{1} = 75 \text { mm, }$ transforms into the far-field and $L_{2} = 300 \text { mm, and } L_{3} = 50 \text { mm} \, (150) \text { mm}$ demagnify this far-field plane of the down-conversion crystal by a factor of 6 (2) onto the photon number resolving (or EMCCD) detector.

The experimental setup for assessing the camera performance is as shown in Fig. 1. The laser pump beam at $355 \text { nm}$ expanded to $\sim 10 \text { mm}$ in diameter, collimated and incident upon a BBO crystal of thickness $3 \text { mm}$ cut for type-II parametric down-conversion at degenerate wavelength of $710 \text { nm}$. Immediately after the down-conversion crystal the remaining pump beam is removed by a pair of dichroic mirrors eliminating the autofluorescence of the subsequent optical components that would otherwise increase background noise.

The down-converted light at $710 \text { nm}$ is selected by a pair of interference filters. The first interference filter $710/10 \text { nm}$ is mounted such that it may be slightly tilted to tune bandpass of filter to the degenerate wavelength, and the second filter with a wider bandwidth $711/25 \text { nm}$ is mounted on the detector to further reduce stray light reaching the detector. The type-II, angle-tuned phase matching combined with the birefringence of the down-conversion crystal results in the signal and idler photons exiting the crystal in slightly different directions such that, in the far-field of the crystal the signal and idler distributions are spatially separated from each other. In the far-field the effect of tilting the $710/10 \text { nm}$ interference filter can be observed and the filter angle is adjusted to obtain equally-sized beams, such that, the full extent of the detected portions of the down-converted beams contain momentum anti-correlated photon-pairs. This far-field plane of the down-conversion crystal is imaged onto the qCMOS detector array for which it is demagnified by a factor of 6, or onto the EMCCD detector, demagnified by a factor of 2. The difference in demagnification between the two cameras is to match the number of down-converted modes to the number of detector pixels in the two experimental configurations for the detectors of differing pixel sizes $4.5 \times 4.5 \, \upmu \text {m}$ on the qCMOS detector and $16 \times 16 \, \upmu \text {m}$ on the EMCCD.

The power of the pump beam incident upon the down-conversion crystal is controlled by rotating a half-wave plate positioned in the pump beam positioned prior to wire grid UV polariser. Large reductions in pump power can be made by inserting an additional neutral density filter into the pump beam prior to the down-conversion crystal. The power of the pump beam in the plane of the down-conversion crystal is measured using a power meter (PM) placed at the exit port of the first dichroic mirror.

### Measurement of the correlation peak

The width of the measured correlation between the signal and idler photon positions in the far-field of the down-conversion crystal arises from the uncertainty in the transverse momentum of the down-converted photon-pairs. This arises from the uncertainty in the transverse momentum, i.e. width, of the pump beam. The strength of transverse momentum correlation in the far-field is given by Eq. (1) as a function of $w_{p}$ the size of the pump beam, $k_{p}$ the wavevector of the pump beam, *f* the focal length of the lens used to relay the photons into the far-field plane^86–88^. This relationship determines the minimum width of the correlation peak^89,90^. However, the actual detected width of the correlation peak may be larger than this theoretical size due to the limited numerical aperture of the optical system, defocus effects, and/or optical aberrations.

1 $$\begin{aligned} \sigma _{p_{x} , p_{y}} = \frac{4 f}{k_{p} w_{p}} \end{aligned}$$

**Figure 2:**
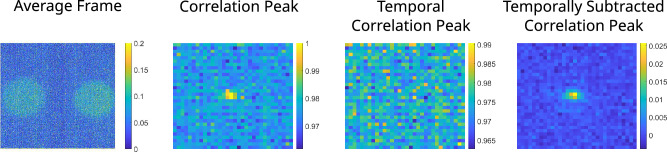
Average Frame and Correlation Peaks. The average frame recorded from 10, 000 frames of average pixel occupancy rate 0.09 events per pixel per frame. Presented alongside are the correlation peak which measures correlations between the photon-pairs and the accidental correlations, the temporal correlation peak recording accidental correlations, and the temporally subtracted correlation peak recording the correlations between the photon-pairs. The correlations were measured on a frame-by-frame basis by taking the cross-correlation of the two regions of interest and averaged for the acquired 10, 000 frames.

When using either an EMCCD or qCMOS camera, the unambiguous detection of a single photon-pair is troublesome since the sensor noise will give a number of false positives, making the true correlation ambiguous. Consequently, it is normal to increase pump power or set the camera exposure time sufficiently high to record multiple photon pairs per frame, allowing the spatial correlation to be observed against a non-zero pedestal. The pedestal arises from random correlations resulting from dark noise and readout noise events from the sensor, stray photons, and losses of either the signal or idler photon. The pedestal is present in the intensity correlation regardless of whether any spatially-correlated photon-pairs are present as it represents the distribution of randomly correlated event pairs. The correlations between the spatially-correlated photon-pairs comprise the correlation peak. In the presence of spatially correlated photon-pairs the correlation peak becomes evident atop the pedestal upon averaging over a sufficient number of frames, dependent on the illumination level and the detector being used. For an illumination level equivalent to an average pixel occupancy rate of 0.09 events per pixel per frame the measured correlation peaks are shown in Fig. 2. The measured pixel occupancy rate is the average number of photon events per pixel per frame across the region of interest that comprises the beams. This is equivalent to the SPDC illumination level, modified by the quantum efficiency of the optical system and the detector. The correlation peak arises from a measurement in which the two regions of interest, corresponding to signal and idler SPDC beams, are from the same frame and hence exhibit strong spatial correlations. This contains both the true correlations between the SPDC photon-pairs and also random correlations that correspond to the pedestal. The temporal correlation peak corresponds to the correlation pedestal and is measured by taking the same two regions of interest but from two subsequent frames for which the true spatial correlations are absent and only the random correlations are present. The temporally subtracted correlation peak is the correlation peak minus the temporal correlation peak thereby removing the pedestal and other features present as a result of background and sensor noises to leave only the true spatial correlations between the photon-pairs. It is from this temporally subtracted correlation peak that the visibility of the correlation is assessed. The overall aim of this work is to compare the number of frames over which the data has to be averaged in order to observe the correlation peak and understand how this averaging number changes depending upon the number of photon-pairs per frame. For our experimental configuration the widths of the correlation peaks on the two cameras were calculated to be equal to $\sigma _{qCMOS, x} = 2.09$, $\sigma _{qCMOS, y} = 1.32$ pixels and $\sigma _{EMCCD, x} = 2.37$, $\sigma _{EMCCD, y} = 1.37$ pixels.

**Figure 3:**
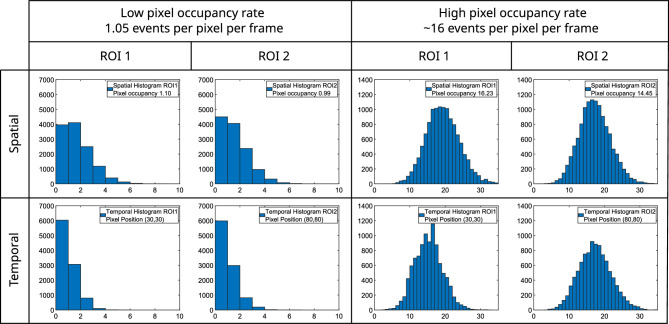
qCMOS data histograms. Spatial histogram of the two regions of interest that comprise the two downconversion beams under a pixel occupancy rate of 1.05 first row in (**a**). Temporal histograms for camera frames for corresponding pair of anti-correlated pixel positions second row in (**a**). Spatial histogram of the two regions of interest that comprise the two downconversion beams under a pixel occupancy rate of 15.34 first row in (**b**). Temporal histograms across a set for camera frames for corresponding pair of anti-correlated pixel positions second row in (**b**). Temporal histograms calculated from 10, 000 frames. ROIs are $111 \times 111$ pixels with centre point (55, 55), meaning pixel positions (30, 30) and (80, 80) are anti-correlated. Data acquired using photon-counting mode of the qCMOS detector.

In Fig. 3 we present spatial histograms of all pixels in the regions of interest of that comprise the two downconversion beams under pixel occupancy $1.05$ events per pixel per frame on average on the first row of the left cell, alongside temporal histograms across a set of $10,000$ camera frames for a corresponding pair of anticorrelated pixel positions from the same dataset obtained using the photon number resolving detector on the second row of the left cell. We also include a set of histograms for a higher level of average pixel occupancy of $\sim 16$ events per pixel per frame with the spatial histogram of all pixels in the region of interest on the first row of the right cell, and temporal histograms for a corresponding pair of anticorrelated pixels on the second row of the right cell. The correlations were measured on a frame-by-frame basis by taking the cross-correlation of the two regions of interest and averaged for the acquired $10,000$ frames per dataset. The form of the histograms presented in Fig. 3 resemble Poissonian distributions because for spontaneous emission as is the case for SPDC there are a low number of photons $<<1$ per spatiotemporal mode and in this regime the Bose-Einstein distribution of the SPDC is indistinguishable from a Poissonian distribution^26,91^.

## Results

To assess the number of frames required to identify the correlation peak across a range of light levels a visibility criteria must be defined. We measure the peak to noise ratio $\text {(PTNR)}$ in the temporally subtracted correlation peak and define the presence of the correlation peak to be when a value of $\text {PTNR} \ge 5$ is reached. Prior to assessing the $\text {PTNR}$, a low-pass filter, defined by a sigma of 0.75 pixels, is applied to the temporally subtracted correlation peak. This low-pass filter is applied so as to smooth the temporally subtracted correlation peak without unduly broadening it while preventing pixels exhibiting high noise from being incorrectly identified as satisfying the $\text {PTNR}$ condition. We apply and compare the data acquired using the photon number resolving qCMOS detector in the photon-counting readout mode to the same data that is binarised as a simulation of a single photon sensitive detector that is unable to distinguish beyond either 0 or $\ge 1$ photons. We then compare this to both binarised and analogue data acquired using the EMCCD detector. For each pixel occupancy level 10, 000 camera frames are obtained.

**Figure 4:**
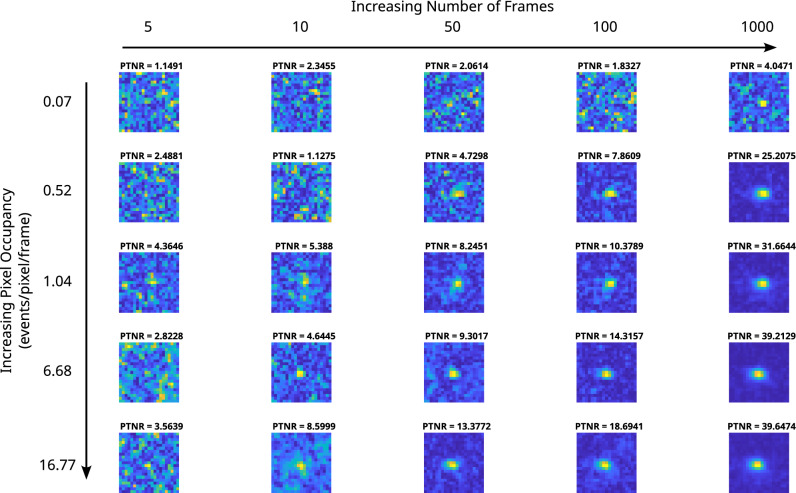
Correlation Peak Array for the qCMOS camera. Here we present a grid of correlation peaks calculated at 5, 10, 50, 100, 1000 frames (left to right) for a range of pixel occupancy rates from 0.07 to 16.77 (top to bottom) events per pixel per frame on average. It can be seen that as the pixel occupancy and/or number of frames increase then fewer frames are required to improve the PTNR and therefore confirm the presence of spatial correlations.

A time-series of correlation peaks from $0-1000$ frames for a range of pixel occupancy rates is shown in Fig. 4 for data acquired using the photon number resolving qCMOS detector. In this figure it can be seen that as the pixel occupancy rate increases the number of frames needed to find a correlation peak that satisfies the condition of $\text {PTNR} \ge 5$ is reduced across all pixel occupancy rates considered here.

**Figure 5:**
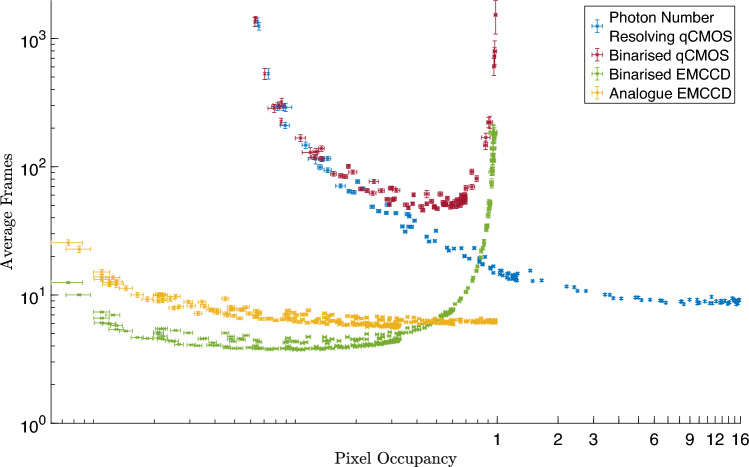
Average number of frames vs pixel occupancy rate. Plot for the average number of frames required to measure a correlation peak with a $\text {PTNR} \ge 5$. The qCMOS detector in photon counting readout mode in blue is capable of observing correlations out to a pixel occupancy rate of $\sim 16$ events per pixel per frame. The binarised qCMOS data in red shows the performance of this detector when following a thresholding scheme for photon numbers $\ge 1$. This is for comparison to the thresholding employed when using the EMCCD camera for which the binarised EMCCD data is presented in green. For both biniarised datasets there is a limit as pixel occupancy rate approaches 1 event per pixel per frame. The corresponding analogue data for the EMCCD camera is presented in orange for comparison.

In Fig. 5 the number of frames required to measure a correlation peak with a $\text {PTNR} \ge 5$ is presented for the qCMOS detector in photon counting readout mode (blue), qCMOS data binarised (red), EMCCD data that is binarised (green), and the corresponding analogue EMCCD data (orange). For the qCMOS detector in photon-counting readout mode the number of frames required to confirm the presence of the correlation peak across a range of light levels decreases from $\sim 1000$ frames to $\sim 10$ frames as the measured pixel occupancy rate increases up to $\sim 16$ events per pixel per frame. Using the same qCMOS detector data but thresholding and binarising the frames it can be observed that as the pixel occupancy rates increases the number of frames required to observe the correlation peak initially decreases until the number of photons per pixel per frame reaches $\sim 0.5$ then levels off before then increasing at around $\sim 0.7$ events per pixel per frame. As the light level continues to increase the number of frames required to observe the peak increases before the $\text {PTNR}$ condition can eventually no longer be met, as the binarised pixel occupancy rate approaches one event per pixel per frame. Binarised data from the EMCCD detector shows that fewer frames are required at lower light levels to observe the correlation peak than in the case of either qCMOS data sets and that there is similar behaviour to the binarised qCMOS data as the pixel occupancy rate approaches one event per pixel per frame. The EMCCD demonstrates an improved performance in terms of the number of frames required to measure the correlation peak across the pixel occupancy rates presented here requiring $\sim 10$ frames for pixel occupancy rates up to $\sim 0.7$ events per pixel per frame for binarised frames. The qCMOS detector reaches comparable performance in terms of the number of frames required but for pixel occupancy rates $>1$ event per pixel per frame whereby it approaches $\sim 10$ frames to meet the $\text {PTNR} \ge 5$ requirement. The EMCCD detector technology is more sensitive and capable of measuring the correlation peak with $\sim 10$ frames under low pixel occupancy rates from $\le 0.1$ event per pixel per frame due to increased sensitivity to single photons and noise performance. As a result of the ability to resolve photon number the qCMOS technology is able to operate at increased pixel occupancy rates $>1$ event per pixel per frame and requires a similar number of frames ($\sim 10$ frames) to measure a correlation peak. The binarised EMCCD data has improved performance over the analogue EMCCD data for pixel occupancy rates $<0.3$ events per pixel per frame. This is because binarisation serves to reject detector noise events and select photon events by thresholding at a level of $\upmu _{Dark} + 3.5\sigma _{Dark}$ for camera readout noise measured from dark frames of mean $\upmu _{Dark}$ and standard deviation $\sigma _{Dark}$. This data is also presented in Table 1.

In terms of the time required to acquire frames the qCMOS detector also presents an advantage in that it can operate continuously whilst the EMCCD data was acquired in blocks of 100 frames to allow the sensor temperature to be stabilised. This additional waiting for sensor cooling and stabilisation results in EMCCD acquisition time of 120.0 s and qCMOS acquisition time of 47.5 s for 1000 frames. This is for a comparable exposure time of $0.058 \text { s}$ for the EMCCD and $0.044 \text { s}$ for the qCMOS. The qCMOS detector is also acquiring a region of dimension $500 \times 500$ pixels compared to $512 \times 512$ for the EMCCD.

**Table 1:** This table presents the average number of frames required to meet the condition $PTNR \ge 5$ split into four pixel occupancy rate ranges shown in Fig. 5 and described in the text.

		Pixel occupancy rate (events per pixel per frame)			
		$0-0.08$	$0.08-0.7$	$0.7-1$	$1-16$
Average number of frames	Photon-number resolving qCMOS	–	$10^3>> 10^2$	$10^2>> 10^1$	$\sim 10$
	Binarised qCMOS	–	$10^3>> 10^2$	$10^2>> 10^4$	–
	Binarised EMCCD	$\sim 10$	$\sim 10$	$10>> 10^3$	–
	Analogue EMCCD	$\sim 10$	$\sim 10$	$\sim 10$	–

**Figure 6:**
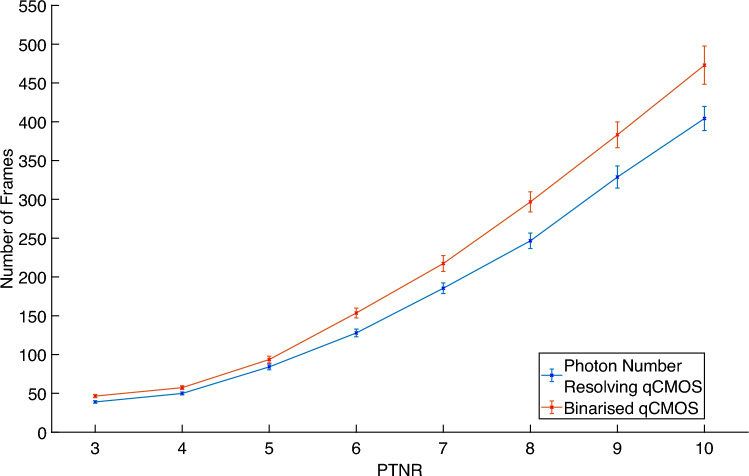
PTNR vs Number of Frames. The average number of frames required to achieve a PTNR across the range of $3-10$ for data representing an average pixel occupancy rate of 0.09 events per pixel per frame as measured using the photon-counting readout mode. The number of frames increases with the square of the increase of the PTNR for both photon-number resolving and binarised frames.

We also investigated the number of frames required to meet a given PTNR condition for a given pixel occupancy rate of 0.09 events per pixel per frame. As displayed in Fig. 6 the number of frames required to meet the given PTNR condition increases with the square of the increase in PTNR as might be expected. This behaviour is present for both photon-counting and binarised frames taken with the qCMOS detector.

## Conclusions

We compare the number of frames required to optimise the correlation peak with the use of a photon-number resolving detector and an EMCCD detector across a range of illumination levels. This is calculated for photon number resolved data and also binarised data for the qCMOS. The EMCCD data is both in the form of analogue and binarised data. We demonstrate that at across the measured occupancy rates the EMCCD detector is capable of measuring the correlation peak using fewer frames than the qCMOS detector due to increased sensitivity and noise performance. In addition to requiring fewer frames, the EMCCD is able to resolve a correlation peak at lower pixel occupancy rates than the qCMOS detector. When the EMCCD is assessed using both analogue and binarised data $\sim 10$ frames are required to meet the condition of $\text {PTNR} \ge 5$. The binarised EMCCD data has an advantage over the analogue EMCCD data up to a pixel occupancy of $\sim 0.7$ events per pixel per frame, at which point the number of frames required to meet the $\text {PTNR}$ condition increases above that of the analogue data. For the qCMOS detector the binarised data follows a similar trend to that of the binarised EMCCD data with the number of frames required to meet the $\text {PTNR}$ condition increasing as the pixel occupancy rate tends towards 1 event per pixel per frame. When using the photon number resolving mode the qCMOS reaches a comparable performance level to that achieved when using the EMCCD at low pixel occupancy rates, requiring $\sim 10$ frames to meet the condition of $\text {PTNR} \ge 5$. Further to comparable performance at these higher pixel occupancy rates of $\gtrsim 1$ events per pixel per frame, the qCMOS detector is capable of photon-number resolving thereby presenting an advantage in this illumination regime. It is possible to observe the correlation peak using analogue EMCCD data, however, the EMCCD does not have the capacity to resolve photon number. In addition to operating at higher detector pixel occupancy rates than the EMCCD, we find the qCMOS detector is 2.5 times faster for a comparable camera exposure time due to readout and cooling requirements of the EMCCD. We note that similar comparison schemes could also be employed to select the optimal readout parameters such as vertical and horizontal shift speeds and gain modes of detectors such as the EMCCD camera that best meet the $\text {PTNR}$ or some other detection condition. Detectors capable of resolving the number of photons such as the qCMOS detector used here may accelerate the transition of quantum enhanced imaging technologies into real-world applications such as quantum microscopes and covert imaging schemes.

## Methods

The laser source used was a JDSU xCyte CY-355-150 Nd:YAG laser with quasicontinuous output at $355 \text { nm}$ with power output of $150 \text { mW}$, a pulse repetition of $100 \pm 10 \text { MHz}$, and a pulse duration of $>10 \text { ps}$. The spatial filter used to collimate and expand the pump beam comprises a $50 \text { mm}$ lens, a $50 \upmu \text {m}$ pinhole, and a $200 \text { mm}$ lens. The down-conversion source was a BBO non-linear crystal cut for type-II down-conversion at degenerate wavelength of $710 \text { nm}$. Down-conversion crystal dimensions $10 \text { mm } \times 10 \text { mm} \times 3 \text { mm}$. The qCMOS camera used was a Hamamatsu ORCA-Quest qCMOS camera cooled to $-20^{\circ }$C, operated on ultra-quiet mode (rms readout noise $0.24e^{-}$) acquiring frames of $500 \times 500$ pixels at $\sim 22 \text { fps}$ with an exposure time of $0.044064 \text { s}$ which defines the temporal resolution of the system. The EMCCD camera used was an Andor Ultra 897 of pixel size $16 \times 16 \, \upmu \text {m}^2$ with $100\%$ pixel occupancy rate. The camera was cooled to $-90^{\circ } \text {C}$ using peltier and water cooling. Optimal acquisition parameters for the EMCCD camera were set as follows: vertical speed $1.7 \, \upmu s$; voltage clock amplitude $+0\text { V}$, horizontal speed $5 \text { MHz}$; EM gain 1000; pre-amplifier gain set to 1; $512 \times 512$ pixel acquisition region; exposure time of $0.058374 \text { s}$. For the given acquisition parameters the shortest exposure time allowable was used to maximise the data rate. Chroma T4551pxt dichroic mirrors with a cutoff wavelength of $455 \text { nm}$ and $98 \%$ transmission at $710 \text { nm}$ were used to remove the pump beam. The tilt adjustable interference filter is a Chroma ET710/10m interference filters, with a $10 \text { nm}$ bandpass with a top-hat profile centred at $710 \text { nm}$ ($99 \%$ efficiency). The fixed interference filter positioned on the camera is a Semrock FF01-711/25 interference filters, with a $25 \text { nm}$ bandpass with a top-hat profile centred at $711 \text { nm}$ ($99 \%$ efficiency).

## Data Availability

All data needed to evaluate the conclusions in the paper are present in the paper. Additional data related to this paper may be requested from the authors.
